# Athymic murine models can underestimate the immune-dependent components of chemotherapy efficacy

**DOI:** 10.3389/fimmu.2026.1863372

**Published:** 2026-09-08

**Authors:** Taha Umair Wani, Hyonok Yoon, Ji-Ye Kim

**Affiliations:** 1 Department of Pathology, Ilsan Paik Hospital, Inje University College of Medicine, Goyang, Republic of Korea; 2 College of Pharmacy, Research Institute of Pharmaceutical Sciences, Gyeongsang National University, Jinju, Republic of Korea

**Keywords:** athymic murine animals, chemotherapy, immunogenic cell death, T-cells, tumor microenvironment

## Abstract

Athymic mice are widely used in preclinical research to study tumor growth, metastasis, and the efficacy of conventional and targeted therapies. However, these animals are profoundly deficient in thymus-derived conventional T cells while retaining B cells and substantial innate immune function, creating a selective blind spot for treatment effects that require adaptive immune amplification. In addition, many modern chemotherapeutic agents act beyond direct tumor-cell-intrinsic cytotoxicity, relying instead on immunogenic cell death (ICD), antigen presentation, and host immune amplification. An intact immune system is essential for the full therapeutic benefit of these chemotherapy drugs, as agents like anthracyclines and oxaliplatin trigger ICD to recruit and prime T lymphocytes, turning localized tumor damage into systemic, durable protection. Conversely, T-cell deficiency shifts chemotherapy-induced effects toward immunosuppression; in athymic models, the absence of cytotoxic T-cells leaves tumor-associated macrophages and myeloid-derived suppressor cells unchecked, creating an immunosuppressive microenvironment that directly contributes to underestimation of the therapeutic efficacy which is ultimately used in intact immunity. From our perspective, while athymic nude models remain valuable for direct tumor-cell responses and establishing primary human xenografts, but may underestimate endpoints requiring conventional T-cell priming, effector function, checkpoint interactions, systemic tumor control, or immunological memory. We recommend that preclinical study designs adopt a context-appropriate model strategy pairing athymic models with syngeneic or humanized mouse systems to accurately capture the full therapeutic potential of host-dependent anticancer therapies.

## 1 Introduction

Athymic mice have been extensively used in preclinical research to evaluate chemotherapeutic efficacy. These mice enable robust tumor engraftment and reproducible assessment of tumor growth inhibition. Importantly, athymic mice represent a defined experimental model characterized by congenital thymic deficiency and the absence of mature T lymphocytes. In this model, chemotherapeutic agents often show patterns of response that differ from those observed in immunocompetent systems. While not universal, this divergence occurs in a significant and mechanistically relevant proportion of cases (1, 2).

Over the past two decades, accumulating evidence has demonstrated that besides tumor-intrinsic cytotoxicity the efficacy of some chemotherapeutic agents depends on immune-mediated mechanisms (3, 4). Agents such as anthracyclines and platinum compounds induce ICD, enhance antigen presentation, and promote adaptive immune activation. In immunocompetent hosts, these immunostimulatory mechanisms contribute to improved therapeutic responses, sustained tumor control, and the development of immunological memory. In contrast, in athymic mice, where adaptive T-cell immunity is absent, these immunogenic agents frequently trigger alternative biological responses that suppress rather than stimulate the tumor immune microenvironment.

In these models the immunogenic agents often achieve this by supporting the maintenance or expansion of regulatory and suppressive innate immune populations, including myeloid-derived suppressor cells and tumor-associated macrophages with tissue-repair or tolerogenic characteristics (5, 6). At the same time, the upregulated immune checkpoint ligands such as PD-L1 suppress innate immune cells instead of suppressing the active antitumor T-cell responses due to the complete absence of effector T-cells. As a result, immune checkpoint signaling functions primarily as a regulator of baseline immune activity rather than as a feedback mechanism restraining active antitumor T-cell responses.

In the following sections we highlight in detail some of the most important aspects of chemotherapy in athymic mice. Relevant literature was identified through searches in Google Scholar and PubMed databases from the year 1980 to 2026. Articles discussing the relationship between chemotherapy efficacy and host immune status, particularly studies involving athymic or T-cell–deficient mouse models, were prioritized. Additional relevant publications were identified through manual screening of references cited in key articles.

This review asks not whether chemotherapy has activity in athymic mice, but which components of chemotherapy benefit are systematically missed when mature T-cell responses are absent. We focus on endpoints most vulnerable to underestimation in athymic hosts, including ICD-dependent amplification, checkpoint synergy, suppressive myeloid remodeling, and immune memory.

## 2 Host immune competence contributes to the efficacy of selected chemotherapy regimens

A growing body of evidence has expanded the classical view of chemotherapy as a purely tumor-cell-intrinsic cytotoxic intervention. In many reviewed models, chemotherapeutic agents derive part of their efficacy from immune-dependent mechanisms involving adaptive antitumor immunity. In the absence of T lymphocytes, this immune-dependent component is often attenuated, limiting the conversion of chemotherapy-induced tumor damage into durable tumor control, antigen-specific recall, and immune memory (7, 8).

**Figure 1:**
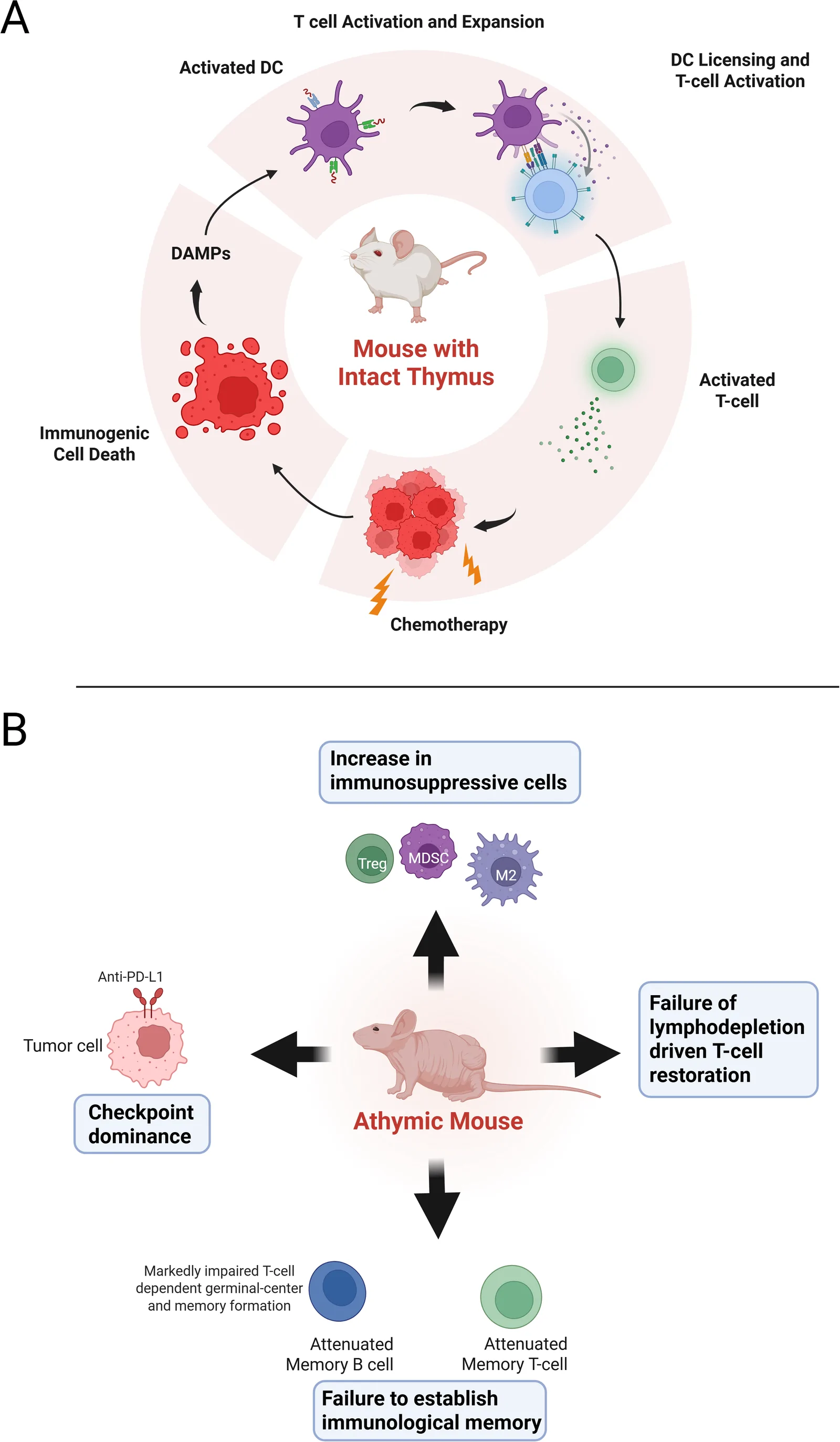
Intact immune system is essential for the full therapeutic benefit of chemotherapy. **(A)** In mice with an intact thymus, chemotherapy-induced ICD releases damage-associated molecular patterns (DAMPs), promoting dendritic cell (DC) activation and licensing, followed by T-cell activation and expansion. This immune amplification can contribute to the antitumor efficacy of chemotherapy. **(B)** In athymic mice, the absence of a functional thymus and mature T-cell compartment limits chemotherapy-induced adaptive immune responses, resulting in increased immunosuppressive cell populations (Tregs, MDSCs, and M2 macrophages), checkpoint-dominant immune regulation, failure to restore lymphodepletion-driven T-cell responses, and impaired establishment of immunological memory. These limitations may lead to an underestimation of the immune-dependent efficacy of chemotherapy in athymic tumor models. This image was created using BioRender.

In many ICD-prone and immunomodulatory regimens, the T-cell-dependent component of chemotherapy benefit is diminished or lost in the absence of mature T lymphocytes (9). Although chemotherapy directly induces tumor cell death, its capacity to convert this cytotoxic stress into effective tumor control depends on T-cell–mediated recognition of therapy-exposed tumor antigens (10). When T-cells are absent, chemotherapy-induced ICD does not translate into productive antigen presentation, clonal expansion of tumor-specific effector T-cells, or the establishment of immunological memory (11). As a result, treatment responses are limited to short-term tumor regression, with no protection against tumor rechallenge and no discrimination between tumor-associated and irrelevant antigens (12). This immune deficiency can blunt durable tumor control, eliminate rechallenge protection, and reduce synergy with immunotherapy. Collectively, these findings indicate that T-cells are often required for the antigen-specific and durable components of chemotherapy benefit, and that their absence can reduce chemotherapy to a predominantly cytotoxic intervention with limited immune reinforcement or memory formation (13). The reviewed literature highlights several functional domains through which an intact immune system contributes to chemotherapy efficacy, as summarized in **Figure 1**.

### 2.1 Host immunity dictates the efficacy of novel drug formulations

Advanced drug delivery systems, including liposomal and nanoparticle-based formulations, are widely used to enhance therapeutic efficacy via improved pharmacokinetics, tumor accumulation, and cellular uptake (14, 15). However, emerging evidence suggests that their effectiveness remains critically dependent on host immune competence, with therapeutic failure frequently observed under conditions of immune deficiency (16).

Some liposomal and nanoparticle formulations appear to derive part of their added benefit from better preservation or recruitment of antitumor immunity in immunocompetent hosts (17). Several reported formulations have been designed to specifically kill tumor cells while causing negligible harm to immune cells (especially CD8+ T-cells). For example, this is demonstrated by oxaliplatin liposomes developed by Shimizu et al. (18), whose surface modification with polyethylene glycol (PEGylation) prevents them from being recognized and cleared by the mononuclear phagocyte system (MPS). This long circulation allows the liposomes to preferentially accumulate in tumor tissues via the enhanced permeability and retention (EPR) effect, resulting in significantly higher intratumoral drug concentrations compared to the free drug (19). Besides direct cytotoxicity and depletion of immunosuppressive populations such as MDSCs and TAMs, these liposomes may help preserve T-cell levels and enhance MHC-I expression. In athymic mice, the depletion of suppressor cells and upregulation of MHC-I provide would be expected to provide limited benefit, as there is no mature CD8+ T-cell population available to capitalize on this primed environment.

Similarly, oxaliplatin nanocarriers by Zhao et al. produced dramatic increases in the release of Damage-Associated Molecular Patterns (DAMPs), calreticulin (CRT) exposure on the cell surface, extracellular ATP secretion, and HMGB1 leading to stronger downstream immune consequences of ICD in Immunocompetent settings (20).

Much of this difference is better attributed to inefficient translation of ICD-associated signals into dendritic-cell activation, T-cell priming, and immune memory. Many drugs rely on ICD for their antitumor action e.g., oxaliplatin and doxorubicin which rely upon T-cells for the final outcome (9). DAMPs released during ICD help activate antigen-presenting cell programs that support cross-priming and downstream T-cell responses (21). Accordingly, an increasing number of studies report reduced efficacy of otherwise effective ICD-inducing anticancer drugs in immunodeficient mice. For instance, while doxorubicin (DX) successfully induces the markers of ICD in both immunocompetent and immunodeficient models, the lack of T-cells in immunodeficient mice reduces its durable antitumor effects [Ref]. Therefore, the drug produces only a modest delay in tumor growth through direct cytotoxicity rather than durable cure or regression.

In addition to inducing ICD, platinum-based chemotherapeutic agents may also enhance antitumor immunity by expanding the repertoire of tumor neoantigens (22, 23). Recent evidence suggests that platinum compounds can promote neoantigen generation through multiple mechanisms, including treatment-induced genomic mutations (24), epigenetic reprogramming that reactivates the expression of otherwise silent genomic elements (25, 26), and the formation of platinum-modified peptides that may be recognized as non-self by the immune system (22). These processes may increase tumor immunogenicity and improve T-cell recognition, thereby complementing the immune-stimulatory effects of ICD. Although the relative contribution of these mechanisms to clinical responses remains under investigation, they provide an additional rationale for combining platinum-based chemotherapy with immunotherapeutic strategies.

### 2.2 Synergy with immune-modulating drugs, including checkpoint blockade

**Figure 2:**
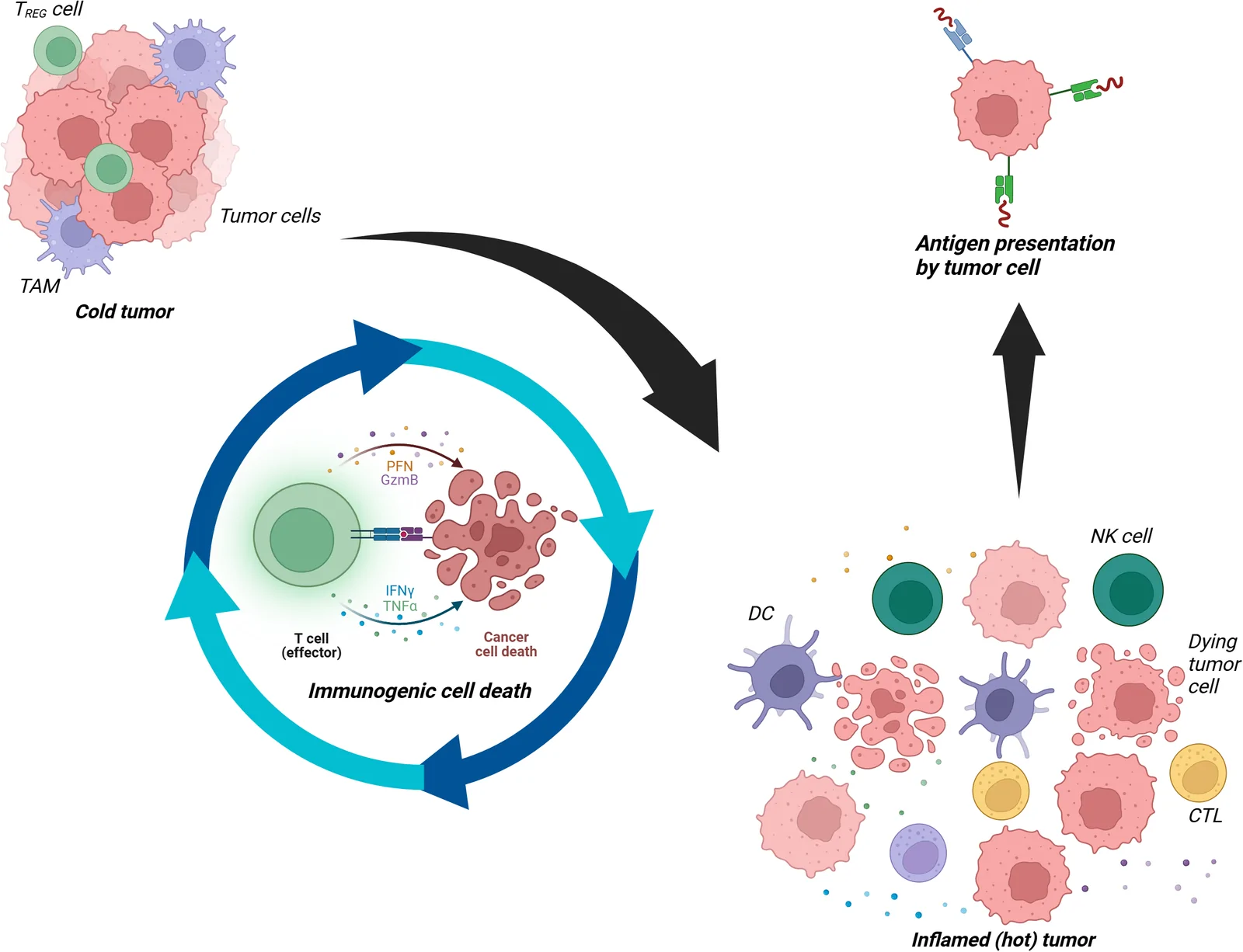
Enhanced antigen presentation by tumor cells. Chemotherapy-induced ICD can convert an immune-cold tumor into a more inflamed state with enhanced antigen presentation and improved responsiveness to immunotherapy. This image was created using BioRender.

Many chemotherapeutic agents show greater activity when combined with immune-modulating drugs. For example, cisplatin exhibits improved antitumor activity when combined with pyridoxine (7), but this benefit is attenuated in immunodeficient settings, supporting an immune contribution to the combination effect. More broadly, synergy between chemotherapy and checkpoint blockade depends on chemotherapy’s ability to convert poorly inflamed tumors into more immunologically inflamed lesions by releasing tumor antigens and DAMPs, promoting dendritic-cell maturation, increasing MHC-I expression, and, in some models, reducing suppressive populations such as Tregs and MDSCs. These changes can enhance T-cell priming and recruitment (**Figure 2**) (27–29), thereby creating conditions in which PD-1/PD-L1 or CTLA-4 blockade becomes more effective. Consistent with this model, oxaliplatin can render prostate tumors more responsive to anti-PD-1 therapy (20, 30), and pegylated liposomal doxorubicin (Doxil) combined with anti-PD-1 or anti-CTLA-4 has produced complete responses and immune memory in immunocompetent models (31). In T-cell-deficient hosts, however, this cooperative mechanism is attenuated or lost because the effector population required for checkpoint blockade to operate is absent.

### 2.3 The immune-dependent efficacy of electrochemotherapy

Electrochemotherapy uses electric pulses to increase drug delivery into tumors (32). The electric pulses increase electropermeabilization of tumor cells and vascular permeability. This strategy has yielded promising results in accumulating drugs in sufficient quantities into the tumor and thus potentiating their anti-tumor efficacy. However, T-cell deficiency can markedly reduce the curative and immune-dependent effects of electrochemotherapy. Sersa et al. compared the anti-tumor effectiveness of ECT with CDDP in immunocompetent C57BL/6 mice versus immunodeficient athymic mice (**Table 1**) (37). In this study by Sersa et al., the immune system appeared essential for achieving complete cures, as immunocompetent mice had significantly longer tumor growth delays and a high percentage of tumor cures, while immunodeficient mice showed no cures. This finding suggests that the host immune response is a critical factor in the complete eradication of tumors following this specific electrochemotherapy protocol.

**Table 1:** Summary of agent-driven tumor mechanisms and immune impacts in athymic models.

Agent/class	Canonical tumor-cell-intrinsic mechanism	Immunostimulatory consequences	Potential immunosuppressive consequences	Predicted limitations of the athymic model	Ref
Anthracyclines (Doxorubicin, Epirubicin, Mitoxantrone)	Topoisomerase II inhibition, DNA intercalation, generation of reactive oxygen species (ROS).	Triggers ICD characterized by ER stress-mediated calreticulin (CALR) exposure, vesicular ATP secretion, HMGB1 release, and cell-autonomous type I IFN signaling with CXCL10 production; recruits CD8\alpha+/CD103+ dendritic cells (DCs) and drives cross-priming of CD8+ cytotoxic T lymphocytes (CTLs).	Myelosuppression at high/maximum tolerated doses (MTD); potential induction of PD-1/PD-L1-dependent immunosuppressive axis and transient MDSC expansion in specific tumor models.	Fails to capture CD8+ T-cell cross-priming, effector CTL killing, helper T-cell activity, memory generation, and synergy with immune checkpoint blockade (e.g., anti-PD-1/PD-L1).	(33, 34)
Platinum Derivatives (Oxaliplatin, Cisplatin/Carboplatin)	Intra- and inter-strand DNA crosslinking, inhibition of DNA replication and transcription.	Oxaliplatin: Promotes bonafide ICD via CALR exposure, ATP release, and HMGB1 secretion, driving systemic T-cell responses. Cisplatin/Carboplatin: Non-ICD inducers natively; downregulate PD-L2 on DCs and upregulate tumor MHC class I/NKG2D ligands.	Oxaliplatin: Can induce recruitment/expansion of immunosuppressive IgA-producing plasma cells or myeloid cells in specific tissue beds. Cisplatin: Recruits immunosuppressive tumor-associated macrophages (TAMs) and promotes M2 polarization.	Fails to model CD8+ T-cell-dependent efficacy of oxaliplatin, T-cell priming, memory formation, and synergy with anti-PD-1/CTLA-4 therapies.	(16, 33–36)
Alkylating Agents (Cyclophosphamide, Melphalan)	DNA crosslinking, destabilization of DNA replication forks.	Metronomic/low doses selectively deplete CD4+CD25+FOXP3+ regulatory T (TREG) cells; induces translocation of gut commensal bacteria to secondary lymphoid organs to drive TH17/TH1 responses; boosts type I IFN-dependent DC cross-priming.	High doses cause severe, non-selective lymphopenia/myelosuppression; acute administration can transiently expand MDSCs and upregulate PD-L1.	Fails to acquire TREG depletion-mediated restoration of effector T-cell proliferation, helper T-cell (TH1/TH17) polarization, and T-cell-dependent long-term immunity.	(16, 33, 34)
Taxanes (Paclitaxel, Docetaxel; Vinca Alkaloids: (Vinorelbine)	Interference with tubulin polymerization/depolymerization, mitotic arrest, polyploidization.	Polyploidy induces ER stress leading to CALR exposure; direct activation of TLR4 on DCs by paclitaxel enhances cross-priming; increases intratumoral CTL/TREG ratio and activates NK cells.	Promotes recruitment of CD68+ TAMs and their M2 immunosuppressive polarization; high-dose administration induces lymphopenia.	Fails to attain CD8+ CTL effector responses, CD4+ helper T-cell activity, and synergy with anti-CTLA-4 or anti-PD-1 checkpoint inhibitors.	(16)
Antimetabolites (5-Fluorouracil, Gemcitabine)	Inhibition of pyrimidine/purine synthesis, incorporation into DNA/RNA causing chain termination.	Selectively depletes circulating and intratumoral MDSCs; reprograms TAMs toward an immunostimulatory phenotype; upregulates tumor MHC class I and NKG2D ligands; increases CTL infiltration.	Depletion of MDSCs via cathepsin B release can trigger NLRP3 inflammasome activation and IL-1\beta secretion, promoting tumor inflammation; combination with irinotecan (FOLFIRI) abolishes MDSC clearance.	Fails to secure CTL effector expansion, TH1 cytokine responses, and the full therapeutic synergy when combined with T-cell-directed immunotherapies.	(16)
Proteasome Inhibitors (Bortezomib)	Inhibition of 26S proteasome activity, accumulation of misfolded proteins, induction of terminal unfolded protein response (UPR).	Induces ICD accompanied by surface exposure of heat shock proteins (HSP70, HSP90) and CALR, driving DC maturation and cross-priming.	Dose-dependent lymphopenia and transient inhibition of primary T-cell activation/proliferation.	Fails to obtain T-cell cross-priming, CD8^+^ effector responses, and memory generation triggered by immunogenic proteasome inhibition.	(16)

Mechanistically, the immune-dependent efficacy of electrochemotherapy (ECT) is primarily driven by the induction of ICD and the subsequent recruitment of systemic adaptive immunity (38, 39). The combination of electric pulses and cytotoxic agents such as bleomycin or cisplatin causes rapid tumor cell destruction accompanied by the massive exposure and release of damage-associated molecular patterns (DAMPs), including surface-exposed calreticulin, extracellular ATP, and high-mobility group box 1 (HMGB1) (38, 40). These DAMPs act as adjuvant signals that activate local antigen-presenting cells, particularly dendritic cells, which take up released tumor-associated antigens and migrate to regional lymph nodes to prime CD8+ cytotoxic T lymphocytes (39, 40). In addition, ECT causes a transient localized disruption of tumor microvasculature, leading to tumor hypoxia and necrosis that further amplifies inflammatory signaling and leukocyte infiltration. This entire cascade effectively turns the primary tumor into an *in-situ* vaccine, generating a systemic, T-cell–mediated antitumor response necessary for complete local eradication and long-term memory protection.

To clarify how tumor-targeted therapies intersect with host immunity, we evaluated these agents through theoretical mechanisms (**Table 1**) and experimental evidence (**Table 2**).

**Table 2:** Individual studies showing immunomodulatory effects of chemotherapeutic agents in athymic mice.

Drug	Experimental model	Dose frequency	Quantitative evidence of immune dependence	Direct efficacy finding	Immune interpretation	Ref.
Cyclophosphamide (50 mg/kg, 5-fluorouracil (50 mg/kg) Oxaliplatin (6 mg/kg)	CT26 and MC38 models - BALB/c mice and C57BL/6 mice	After tumor growth, two i.p. injections 8 days apart	Triple combination in CT26 or MC38 mice 1. significantly decreased tumor volume than in athymic mice 2. increased CD4^+^ and CD8^+^ cells at tumor site and in spleen	Tumor volume was reduced in immunocompetent hosts relative to athymic hosts	Depletion of suppressive myeloid/Treg compartments is insufficient without T-cell-mediated clearance of residual tumor.	(41)
Cyclophosphamide	MH129 hepatoma; C3H/HeN and BALB/c nude (nu/nu) mice	20 or 200 mg/kg i.p.	Both 20 and 200 mg/kg cyclophosphamide injected 7 days after tumor cell inoculation significantly decreased the volume of MH129 tumors in C3H/HeN mice. Low-dose cyclophosphamide had no effect on tumor volume in athymic mice Low dose selectively depleted CD4+CD25+ T-cells in C3H/HeN mice High-dose cyclophosphamide was equally effective in both mice Low-dose cyclophosphamide showed higher intratumoral lymphocyte infiltrations	Antitumor efficacy was attenuated in athymic hosts.	Treg-targeting and immunostimulatory effects require functional T-cells for full tumor eradication.	(42)
Cyclophosphamide	Lymphoma L-TACB: Female IIM *e*/Fm rats and N:NIH (S)-nu athymic mice	10 mg/kg, three times per week	Tumor volume decreased in rats but not in athymic mice after the Cy treatment		Absence of the adaptive immune response impairs, at least in part, the therapeutic antitumor effect of MCT with Cy in the L-TACB lymphoma model	
Cyclophosphamide	MethA Fibrosarcoma; BALB/c vs athymic mice			Therapeutic benefit was reduced in athymic hosts relative to immunocompetent mice.	Immunomodulatory benefit of cyclophosphamide is blunted without an adaptive T-cell compartment.	(43)
Cyclophosphamide Doxorubicin	CT26 Colorectal Carcinoma; BALB/c and BALB/c nu/nu female mice	Cy 100 mg/kg, i.p. on day 10 DR 120 ug/40ul, i.t. on days 12, 14, and 16	The i.p. injection of CP alone suppressed the tumor volume of bilateral CT-26	Combination efficacy was attenuated in athymic hosts compared with immunocompetent mice.	ICD plus Treg depletion does not translate into full benefit without T-cell priming and effector function.	(44)
Electrochemotherapy + Cisplatin	LPB Sarcoma; C57Bl/6 and immunodeficient Swiss nu/nu mice (nude)	Cisplatin (1, 4, 6 and 8 mg kg^-1^) Electric pulses using two parallel stainless steel plate electrodes 6 mm apart. Eight squarewave high-voltage dc pulses (amplitude 780 Y, pulse width 100 μs, repetition frequency I Hz)	Tumor volume decreased with combination therapy in immunocompetent mice but not in immunodeficient Increasing CDDP doses + electrochemotherapy, proportionally cured higher percentages of the tumors in C57BI/6 mice No cures were observed in immunodeficient athymic mice regardless the CDDP dose used	Immunocompetent mice showed markedly longer tumor-growth delay and tumor cures, whereas athymic hosts showed no cures.	Durable benefit after electrochemotherapy requires host immune participation beyond enhanced drug delivery alone.	(37)
Oxaliplatin + Cisplatin	CT26 Colorectal Carcinoma; BALB/c (H-2d), C57BL/6 (H-2b) and nu/nu BALB/c mice	Not specified	Both oxaliplatin and cisplatin induce immunogenic colon cancer cell death CT26 tumors implanted in immunocompetent mice reduced in size with OXP treatment and this therapeutic response was lost in immunodeficient mice.	ICD-associated tumor death did not translate into full therapeutic benefit in athymic hosts.	CRT-linked ICD requires downstream dendritic-cell/T-cell coupling for maximal efficacy.	(45)

We summarized the tumor-cell-intrinsic pathways, downstream immune consequences, and inherent limitations of athymic models across major therapeutic classes (**Table 1**). While many agents trigger beneficial innate immune signaling, they frequently induce counter-regulatory immunosuppressive feedback. Crucially, because athymic nude models lack functional T-cells, they cannot capture adaptive immunomodulatory cascades, creating a major translational blind spot for T-cell-dependent agents.

To complement this theoretical framework, we compiled study-specific empirical data across various experimental models, dosing regimens, and quantitative endpoints (**Table 2**). These findings illustrate the degree to which direct anti-tumor efficacy relies on an intact immune system versus direct tumor cytotoxicity, highlighting how model selection directly shapes therapeutic interpretation. Supportive evidence from other T-cell-depleted or immune-ablated murine models and broader or mixed immunodeficient models is summarized in **Supplementary Tables 1**, **S2**, respectively.

## 3 T-cell deficiency shifts chemotherapy-induced effects toward immunosuppression

A recurring pattern in T-cell-deficient settings is that chemotherapy-associated damage is not efficiently converted into productive adaptive immunity and may be accompanied by suppressive myeloid remodeling. Notably, many chemotherapeutic agents widely regarded as immunostimulatory in immunocompetent settings instead exert predominantly immunosuppressive effects in the absence of functional T-cells. Based on an extensive analysis of the literature, we delineate the key mechanisms underlying this context-dependent shift in therapeutic outcome.

### 3.1 Dominance of regulatory and suppressive immune populations

Immunosuppressive cells such as Tregs, MDSCs, and M2-like Tumor-Associated Macrophages (TAMs) play a crucial role in maintaining the immune homeostasis by suppressing excessive immune responses and promoting immune tolerance (46, 47). Such regulatory mechanisms are essential for resolving inflammation and limiting autoimmunity in healthy tissues; however, their excessive accumulation can create a strongly immunosuppressive environment that impairs normal immune function, particularly in cancer (48–50). Within the tumor microenvironment such profound immunosuppressive milieu hinders anti-tumor immunity and frequently impedes therapeutic efficacy, thus facilitating tumor progression. Accordingly, multiple therapeutic strategies have been developed to directly eliminate or re-educate these cells. Well-known agents that can deplete MDSCs include gemcitabine and 5-fluorouracil (5-FU). Wang et al. showed that by reducing MDSC levels, these drugs indirectly decreased Treg recruitment, as MDSCs are a primary source of the chemokines (e.g., CCL3, CCL4, CCL5) that pull Tregs into the area (51). Cyclophosphamide, in low doses, can selectively deplete Tregs without severely damaging other immune cells, helping to break the suppressive cycle. Docetaxel can also reduce MDSC levels and polarize the remaining cells toward a more pro-inflammatory, M1-like state. Contrary to this, chemotherapy on many occasions can also increase the accumulation of immunosuppressive cells in the tumor. Chemotherapy-induced chronic inflammation and tissue damage (e.g., cisplatin) has been shown to drive an influx of MDSCs and M2-polarized macrophages. In some contexts, such as liver cancer, 5-FU has been shown to trigger compensatory MDSC accumulation, which may impair the efficacy of subsequent immunotherapies, including PD-L1 blockade (5). Chemotherapy often reduces Tregs only transiently. Once the drug is cleared, Tregs may recover faster than effector T-cells, thereby favoring tumor relapse.

One of the important determinants of immunosuppressive cell accumulation is the presence or absence of T-cells. Under immunocompetent conditions, T-cells can help counter immunosuppressive stress by entering the tumor site and contributing to the clearance of suppressive cell populations. Immunosuppressive cells may or may not depend directly on T-cells for their function but T-cell absence certainly alters their accumulation within the tumor microenvironment.

#### 3.1.1 Chemotherapy often expands or stabilizes MDSCs in T-cell absence

Although chemotherapy can influence MDSC accumulation independently of T-cells, MDSC-promoting signals often dominate when T-cells are absent. Activated CD8^+^ and Th1 CD4^+^ T-cells normally restrain MDSCs via IFN-γ–mediated differentiation, cytotoxic elimination, competition for cytokines (IL-2, IL-7). In the absence of functional T-cells, the tumor continues to secrete GM-CSF, G-CSF, IL-6, VEGF, CXCL1/2, which drive rapid proliferation and expansion of immature myeloid cells that develop into MDSCs. In such conditions, chemotherapy plays a complex role. While it can reduce the overall “burden” of immunosuppressive cells like MDSCs, drugs like cyclophosphamide and doxorubicin can also paradoxically trigger the release of inflammatory mediators (e.g., GM-CSF, G-CSF, IL1β, IL6) which promote MDSC recruitment.

#### 3.1.2 Chemotherapy reprograms TAMs toward suppressive or repair phenotypes

**Figure 3:**
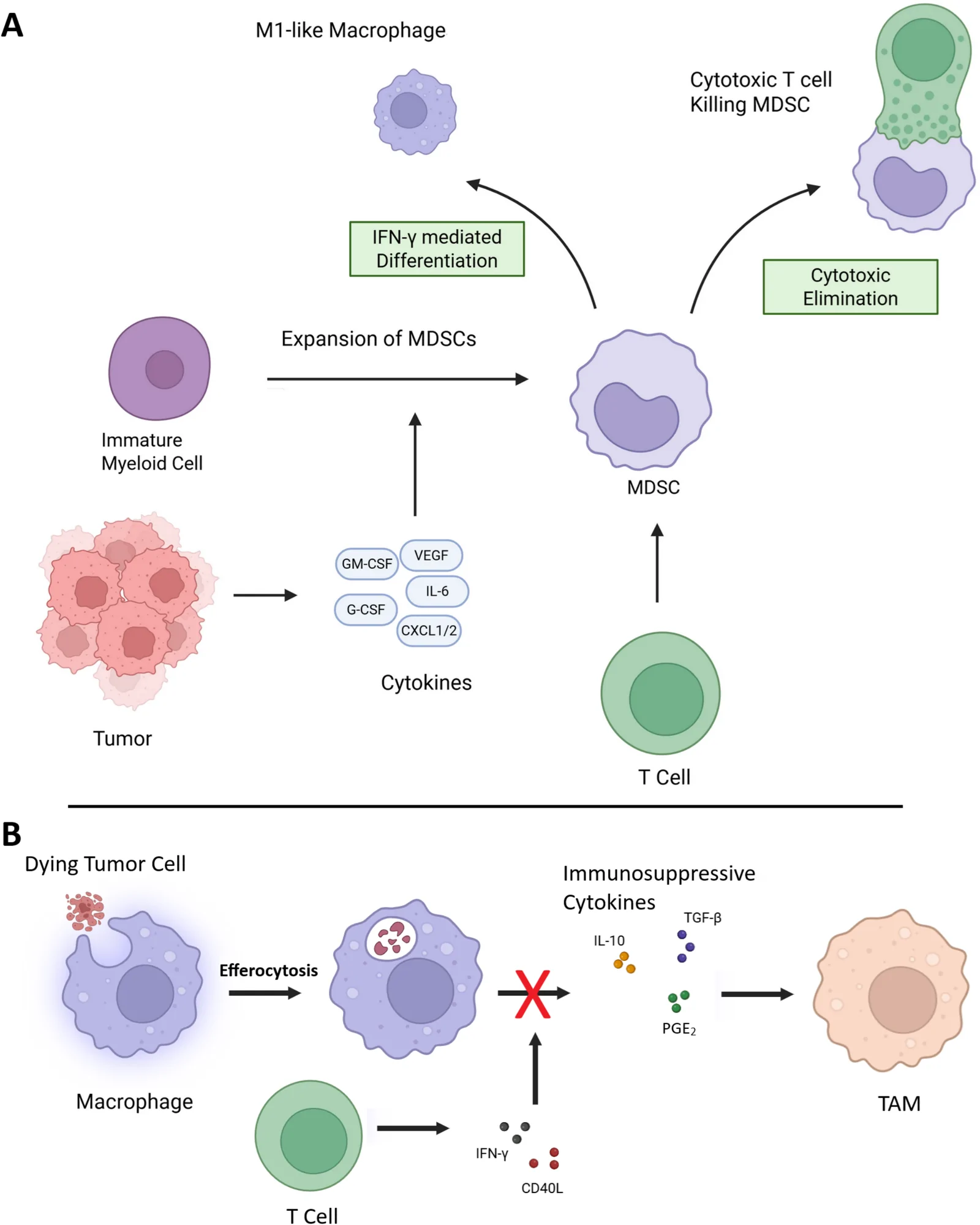
Role of chemotherapy in the expansion of tumor suppressor cells in the absence of T-cells. **(A)** Absence of T-cells leads to the rapid proliferation and expansion of immature myeloid cells into MDSCs which are otherwise restrained by T-cells, **(B)** Chemotherapy leading to efferocytosis and the secretion of immunosuppressive cytokines by macrophages that facilitates TAM production. This image was created using BioRender.

Chemotherapy can induce profound reprogramming of tumor-associated macrophages (TAMs), but the direction of this reprogramming critically depends on signals provided by T-cells. Chemotherapy induces phagocytosis of apoptotic tumor cells, a process known as efferocytosis. Efferocytosis itself promotes anti-inflammatory signaling through mediators such as IL-10, TGF-β, and PGE_2_, which are hallmark features of immunosuppressive and tissue-repair TAM phenotypes (52, 53). These effects are normally countered by classical pro-inflammatory macrophage activation (sometimes referred to as an “M1-like” state) stimulated by interferon-γ (IFN-γ) and contact-dependent signals (e.g., CD40–CD40L) typically provided by T-cells. In athymic mice, without T-cell-derived inflammatory signals (e.g., IFN-γ, CD40L), these macrophages tend to fail to polarize toward pro-inflammatory phenotypes. Instead, they produce immunosuppressive and tissue-repair factors (IL-10, TGF-β, PGE_2_) and differentiate into suppressive TAMs (6). These newly recruited monocytes differentiate preferentially into immunosuppressive TAMs rather than inflammatory macrophages in the absence of T-cell signals. **Figure 3** illustrates the chemotherapy induced immunosuppression in the absence of T-cells in athymic mice.

### 3.2 Long-term immunosuppression instead of immune rejuvenation upon lymphodepletion

**Figure 4:**
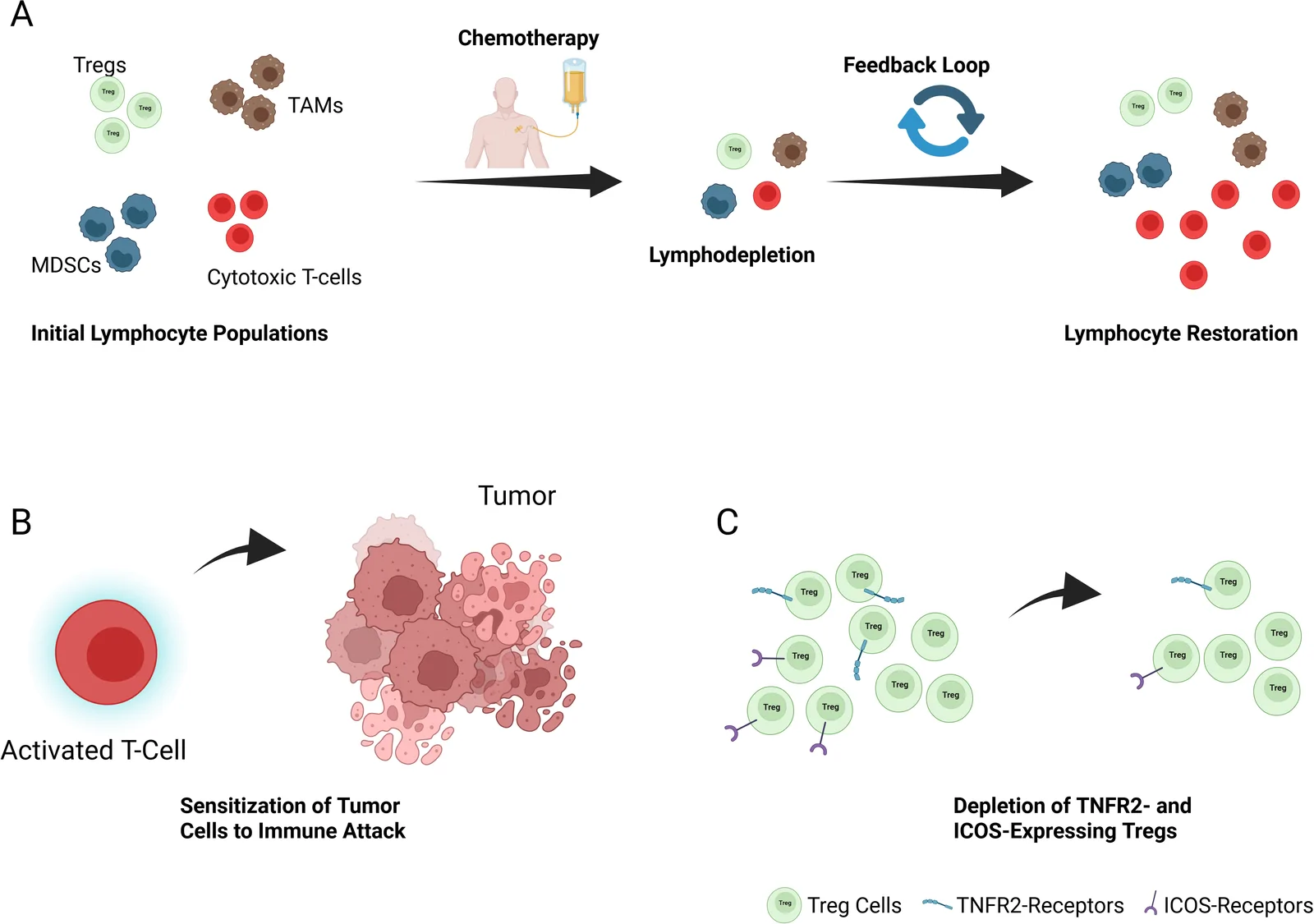
Mechanisms of chemotherapeutic rejuvenation of immune cells. **(A)** Lymphodepletion caused by chemotherapy (e.g., cyclophosphamide) which triggers a feedback loop boosting cytotoxic T-cell restoration while keeping suppressor immune cells under check, **(B)** Sensitization of tumor cells by chemotherapy (e.g., cyclophosphamide) to immune attack, **(C)** Chemotherapy (e.g., cyclophosphamide) induced depletion of TNFR2- and ICOS-expressing Treg cells. This image was created using BioRender.

Certain conventional chemotherapies (like cyclophosphamide and fludarabine) are used to induce transient depletion of endogenous lymphocyte populations of patient’s own immune system. This reduces competing lymphocytes and promotes removal of immunosuppressive cells, release of homeostatic cytokines, enhanced antigen presentation, and remodeling of the tumor microenvironment. Drugs like cyclophosphamide have their efficacy tied to the ability to selectively target cycling TNFR2-expressing regulatory T-cells (Tregs). This creates an environment favorable for the expansion of effector immune cells and boosting immune response. The human immune system remarkably recovers its T-cell populations after being depleted by treatments like chemotherapy or stem cell transplants (54). **Figure 4** presents the schematic of the mechanisms of immune rejuvenation. Reconstituting T-cell immunity is a slow process that often leaves patients vulnerable to infections, disease relapse, and graft-versus-host disease (GVHD) (55). Several cytokines and growth factors that drive these processes include IL-7, IL-15, TGFβ, and KGF. However, paradoxically, this lymphodepletion offers a unique opportunity for immunotherapy because it reduces “cytokine sinks” (cells that consume IL-7 and IL-15) and depletes suppressive Tregs, creating an optimal environment for vaccines and adoptive T-cell transfers to target tumors.

Some chemotherapies enhance the immune response not by creating more immune cells, but by sensitizing tumor cells to attack. Therefore, even during the reduction of immune cells due to overall cytotoxicity, such drugs are still able to kill the tumor cells. Cyclophosphamide (CY) is one example, beyond causing DNA damage, it can sensitize tumor cells to CD8^+^ T-cell attack. In a study by Most et al. cyclophosphamide has been shown to prime tumor cells for immune-mediated elimination by sensitizing them to TRAIL (TNF-related apoptosis-inducing ligand) dependent apoptosis, thereby amplifying subsequent cytotoxic T-cell attack (56). They showed that at the tumor cell level, cyclophosphamide enhances responsiveness to TRAIL through modulation of the extrinsic apoptotic machinery, including upregulation of death receptors DR4/DR5, suppression of inhibitory regulators such as c-FLIP and IAPs, and facilitation of caspase-8 activation. This pharmacological priming converted the otherwise TRAIL-resistant tumor cells into permissive targets. Consequently, activated CD8^+^ T-cells, which express or release TRAIL upon antigen recognition, could engage these sensitized tumor cells more effectively, triggering robust apoptotic signaling independent of or complementary to perforin–granzyme pathways.

Another immunomodulatory mechanism is the selective depletion of specific Treg subsets, for example TNFR2- and ICOS-expressing Tregs targeted by CY (57). TNFR2 and ICOS are key costimulatory receptors that play crucial roles in regulating the immune response, particularly in the function and proliferation of regulatory T-cells (Tregs) and effector T-cells (Teffs). In a study, Most et al. showed that the high TNFR2 and ICOS expression contributed to the highly immunosuppressive phenotype of Tregs. CY was highly effective in depleting TNFR2 and ICOS expressing Tregs (Ki-67hi CD4+ T-cells) which contributed to its superior antitumor action. However, in athymic mice, the T-cell compartment including Tregs is deficient, so the Treg-depleting component of CY activity is diminished.

Therefore, these effects depend on the presence of T-cells and without a functional thymus, lymphodepletion may lead to persistent ineffective immunity and long-term immunosuppression rather than a rejuvenated antitumor response. The absence of thymus also causes the failure of T-cell reconstitution pathways after lymphodepletion. Two non-redundant pathways contribute to post-depletion recovery: thymopoiesis, which generates naïve, antigen-diverse T-cells, and homeostatic peripheral expansion (HPE), which amplifies pre-existing T-cell clones. The absence of a functional thymus (or pre-existing T-cells) substantially limits thymopoiesis (e.g., in athymic mice), forcing immune reconstitution to rely predominantly on HPE. Instead of new, diverse T-cells, the body simply makes more copies of the existing, often exhausted or skewed, T-cell pool. Rather than achieving immune rejuvenation, reconstitution following lymphodepletion results in a quantitatively restored but functionally compromised immune system characterized by functional senescence, limited clonal diversity, and persistent immunosuppression.

### 3.3 Failure to establish immunological memory

**Figure 5:**
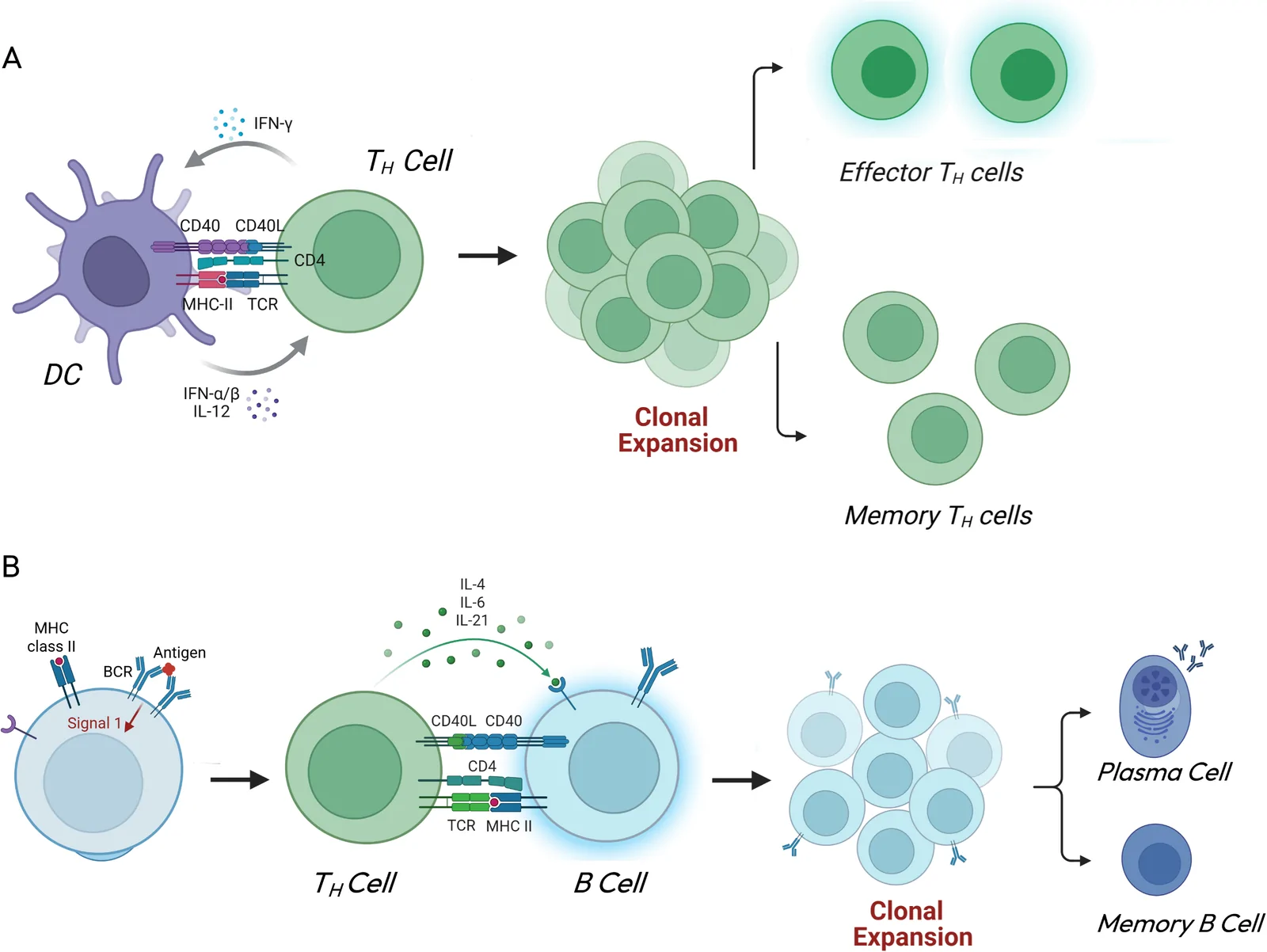
Presence of T-cells is important for immunological memory. **(A)** DC licensing followed by clonal expansion of T-cells and differentiation to effector and memory T-cells, **(B)** Antigen recognition by B cell induces expression of effector molecules by T-cell, activating the B cell leading to clonal expansion of B cells and differentiation to resting memory cells and antibody-secreting plasma cells. In absence of T-cells, memory cell pool fails to form or is severely depleted. This image was created using BioRender.

The adaptive immune system relies on coordinated interactions among T lymphocytes, B lymphocytes, and antigen-presenting cells (APCs) to establish long-term immunity (**Figure 5**). In athymic hosts, absence of T-cells markedly impairs the generation of durable cellular and humoral memory. This section delineates the cellular and molecular mechanisms by which athymia severely impairs conventional T-cell memory and T-cell-dependent humoral memory, although retained B cells may still support selected T-cell-independent antibody responses.

In T-cell-deficient states, particularly when CD4+ T-cells are absent, dendritic-cell licensing and downstream priming are impaired. Lutz et al. showed that CD8+ T-cells activated by unlicensed DCs often undergo anergy (functional unresponsiveness) or apoptosis shortly after activation (58). Even if CD8+ T-cells expand initially, they lack the transcriptional programming (e.g., upregulation of *Bcl-2* and *Bcl-xL*) required to survive the contraction phase of the immune response. This phenomenon produces “helpless” CD8+ T-cells that mount a primary response but fail to differentiate into central memory (T_CM_) or effector memory (T_EM_) subsets.

The generation of high-affinity, class-switched antibodies is the hallmark of humoral memory. This process is strictly dependent on the interaction between antigen-specific B cells and T Follicular Helper (Tfh) cells within the germinal centers (GCs) of secondary lymphoid organs as shown by Thornhill et al. (59). These authors report that in the absence of T-cells, this axis is severed at the co-stimulatory checkpoint. Under normal physiological conditions, Tfh cells provide signals to B cells through the interaction of CD40 ligand (CD154) on the T-cell with CD40 on the B-cell surface. This interaction is critical for the induction of Activation-Induced Cytidine Deaminase (AID), the enzyme responsible for two key processes. The first is the class switch recombination (CSR), switching antibody production from low-affinity IgM to potent IgG, IgA, or IgE isotypes. The second is the somatic hypermutation (SHM), introducing mutations into the antibody variable regions to increase affinity for the antigen.

### 3.4 A proposed checkpoint-rich, effector-poor state in T-cell-deficient hosts

This section explains that while chemotherapy upregulates PD-L1 expression, the presence of T-cells counteracts this effect, a regulatory dynamic that is absent in athymic hosts.

A useful working model in athymic mice is the emergence of a checkpoint-rich, effector-poor microenvironment after chemotherapy. In this setting, cytotoxic stress can increase expression of inhibitory ligands, particularly PD-L1, on tumor cells and innate immune populations through DNA-damage, ER-stress, and interferon-related signaling pathways, even in the absence of T-cell-derived input (35, 60, 61). For example, Jurjen et al. showed that chemotherapy-exposed plasmacytoid dendritic cells (pDCs) displayed impaired STAT1 and STAT3 phosphorylation, attenuating type I interferon amplification, costimulatory molecule upregulation, and cross-priming capacity. Rather than maturing into immunostimulatory antigen-presenting cells, these pDCs adopted a regulatory phenotype in which antigen presentation is uncoupled from productive immune activation. In the absence of T-cells, PD-L1 expression on such cells is not counterbalanced by effector engagement but instead may increasingly favor IL-10, TGF-β, lipid mediators, and other tolerogenic programs and stabilize a suppressive, self-reinforcing immune configuration (62, 63).

The role of effector T-cells in chemotherapy was also demonstrated by Horton et al. These authors attribute the checkpoint inhibitor resistance in non-small cell lung cancer (NSCLC) to a lack of T-cell differentiation into effector CD8^+^ T-cells despite high PD-L1 expression.

This framework suggests that checkpoint pathways in athymic hosts do not simply become irrelevant (64, 65); rather, they may remain prominent features of the tumor microenvironment while being poorly connected to productive antitumor immunity. The result is a checkpoint-rich but effector-poor state that may help explain why chemotherapy-induced PD-L1 upregulation or other immune changes in T-cell-deficient models does not reliably predict durable therapeutic benefit.

Taken together, this proposed model describes a checkpoint-rich, effector-poor state in athymic mice in which inhibitory ligands remain prominent, antigen presentation is only weakly coupled to productive immunity, and cytotoxic therapy may yield transient tumor control without durable immune reinforcement. In this setting, checkpoint induction is not necessarily irrelevant, but its biological meaning differs from that in T-cell-replete hosts and should be interpreted cautiously.

## 4 Perspective

### 4.1 Context-appropriate use of athymic nude models (addressing direct cytotoxicity)

Thymic athymic mice remain highly appropriate for evaluating direct tumor-cell-intrinsic cytotoxicity, pharmacokinetics, and initial drug-dosing dynamics. This model is particularly suitable for non-immunogenic chemotherapeutic drugs (or non-ICD-inducing regimens), high-dose/maximum tolerated dose (MTD) schedules aimed at immediate debulking, and initial screening of human xenografts (66, 67). In these specific contexts where therapeutic outcomes are primarily driven by direct DNA damage or mitotic arrest rather than T-cell priming, athymic models provide a reliable, highly reproducible platform without the confounding effects of variable baseline adaptive immunity.

### 4.2 Limitation contexts (when athymic models compromise efficacy evaluation)

Conversely, athymic models systematically underestimate efficacy when evaluating agents whose mechanism relies on T-cell-mediated reinforcement. This includes ICD-inducing drugs (e.g., anthracyclines, oxaliplatin), low-dose metronomic schedules designed to relieve Treg-mediated suppression, electrochemotherapy, and combinations with immune checkpoint inhibitors or targeted formulations (e.g., liposomes). In these settings, the absence of mature T-cells prevents the translation of drug-induced DAMP release and antigen presentation into durable adaptive responses or long-term immunological memory, causing effective regimens to look only transiently inhibitory.

Chemotherapy agents combined with checkpoint blockers (anti-PD-1/PD-L1, anti-CTLA-4) might show inaccurate results in athymic mice (68). These include that transform “cold” or immune-excluded tumors into “hot” immunogenic lesions by priming T-cells and releasing tumor antigens to facilitate clinical responses by checkpoint blockers.

Discrepancies in drug activity between immunocompetent and athymic models are particularly prominent at lower doses or metronomic schedules (42, 69). Because metronomic dosing often relies on stimulating the immune system rather than direct mass cytotoxicity, these regimens may appear ineffective in athymic models, potentially leading to the premature dismissal of a clinically viable strategy (34, 70).

### 4.3 Considerations and recommendations for preclinical study design

To improve the translational reliability of preclinical oncology studies, researchers should adopt a stratified experimental workflow that strategically incorporates complementary mouse models based on drug class, treatment schedule, and intended mechanisms of action. Rather than viewing athymic nude models as universally flawed, preclinical study designs should apply them deliberately where their strengths lie while complementing them with immunocompetent and selective depletion models to capture immune-dependent efficacy.

To capture the full spectrum of chemotherapy efficacy, preclinical protocols should follow a multi-model paradigm:

Stage 1 (*In Vitro* & Athymic Xenografts): Establish baseline direct cytotoxicity, target engagement, and dosing parameters in human tumor xenografts using athymic nude or severely immunodeficient (e.g., NSG) mice.
Stage 2 (Immunocompetent Syngeneic Models): Complementarily assess immune-dependent therapeutic benefit, durable cure rates, and long-term rechallenge protection in syngeneic immunocompetent models (e.g., CT26, MC38, B16).
Stage 3 (Selective Depletion for Mechanism Dissection): To precisely delineate T-cell-dependent from T-cell-independent components, run parallel cohorts in immunocompetent mice subjected to selective antibody depletion (e.g., anti-CD4, anti-CD8, or anti-Thy1.2). Comparing these depleted cohorts directly against intact syngeneic controls estimates the causal contribution of selected T-cell subsets without the compensatory innate immune hyper-reactivity (such as elevated NK activity) often present in congenitally athymic hosts.

## 5 Conclusion

The growing recognition that the therapeutic efficacy of many chemotherapeutic agents depends partly on host immune responses highlights the importance of selecting appropriate preclinical models. Future studies should evaluate chemotherapeutic agents across complementary experimental systems rather than relying on a single animal model. Immune-competent syngeneic models are essential for assessing the contribution of adaptive and innate immune responses to therapeutic efficacy, whereas T-cell-depleted models can help distinguish T-cell-dependent from T-cell-independent antitumor mechanisms. In addition, immunodeficient models, including nude and NSG mice, remain valuable for investigating direct cytotoxic effects on tumor cells while minimizing the influence of adaptive immunity. Integrating findings from these complementary models will enable a more comprehensive understanding of chemotherapy-mediated antitumor activity, facilitate mechanistic interpretation, and improve the translation of preclinical findings into clinical applications.
