# Supplementary material for: Athymic murine models can underestimate the immune-dependent components of chemotherapy efficacy
Source: Front Immunol. 2026 Sep 8;17:1863372. doi: 10.3389/fimmu.2026.1863372 (PMC13595575; doi:10.3389/fimmu.2026.1863372)
Supplement: Supplementary file 1 [file Table1.docx]

Supplementary Table S1. Evidence hierarchy comparing chemotherapy efficacy in other T-cell-depleted or immune-ablated mouse models.

| **Regimen** | **Model comparison** | **Direct efficacy finding** | **Immune interpretation** | **Ref.** |
| --- | --- | --- | --- | --- |
| Crizotinib + Cisplatin | EML4-ALK Lung Carcinoma; immune-replete vs CD8⁺ T-cell-depleted mice | Combination antitumor activity was reduced after CD8⁺ T-cell depletion. | ICD-associated DAMP release requires CD8⁺ effector engagement for maximal therapeutic benefit. | ^1^ |
| Cyclophosphamide | BNL 1ME A.7R.1 Hepatocellular Carcinoma; immune-replete vs CD4⁺/CD8⁺ T-cell-depleted mice | Tumor control and tumor-specific immune effects were attenuated after T-cell depletion. | Cyclophosphamide-induced antitumor immunity depends on intact adaptive T-cell responses. | ^2^ |
| Cyclophosphamide | P815 Mastocytoma; immune-replete vs thymectomized/irradiated mice | Sustained therapeutic response was weakened in T-cell-depleted hosts. | Adaptive immunity contributes to maintenance of cyclophosphamide-mediated tumor control. | ^3^ |
| Cyclophosphamide | Murine Sarcoma models; immune-replete vs anti-lymphocyte-serum-treated mice | Post-treatment protection and durable control were reduced after lymphocyte depletion. | Host lymphocytes are required for immune memory after cyclophosphamide therapy. | ^4^ |
| Cyclophosphamide | MCA Sarcoma; immune-replete vs whole-body-irradiated / T-cell-depleted mice | Eradication of residual disease was impaired in immune-ablated hosts. | Direct cytotoxicity is insufficient without immune clearance of residual tumor. | ^5^ |
| Cyclophosphamide + Interleukin-2 | B16 Melanoma; immune-replete vs T-cell-immunodepressed mice | Combination outcome depended on intact T-cell function and was not reproducibly beneficial under T-cell-immunodepressed conditions. | IL-2–based modification of cyclophosphamide response depends on a functional T-cell compartment. | ^6^ |
| Cyclophosphamide + Mafosfamide | L1210 Leukemia; immune-replete vs thymectomized/irradiated mice | Augmented therapeutic benefit was reduced in T-cell-deprived hosts. | Alkylator-mediated tumor death requires an immune clearance phase for full effect. | ^7^ |
| Cyclophosphamide + Oxaliplatin | CT26 and MC38 Colorectal Carcinomas; immune-replete vs CD8⁺ T-cell-depleted mice | Combination efficacy was reduced after CD8⁺ T-cell depletion. | ICD plus Treg targeting requires CD8⁺ T-cell priming and amplification. | ^8^ |
| FOLFOX Chemotherapy | CT26 and MC38 Colorectal Carcinomas; immune-replete vs CD8⁺ T-cell-depleted mice | FOLFOX activity and checkpoint-blockade synergy were reduced after CD8⁺ T-cell depletion. | Oxaliplatin/5-FU immune remodeling depends on CD8⁺ T-cell rescue and effector function. | ^9^ |
| Trifluridine/Tipiracil + Oxaliplatin | CT26 Colorectal Carcinoma; immune-replete vs CD8⁺ T-cell-depleted mice | Therapeutic improvement was attenuated after CD8⁺ T-cell depletion. | ICD plus DNA-damage–driven immune modulation is insufficient without T-cell priming and memory. | ^10^ |

Supplementary Table S2. Evidence hierarchy comparing chemotherapy efficacy in non-mouse or mixed immunodeficient models

| **Regimen** | **Model comparison** | **Direct efficacy finding** | **Immune interpretation** | **Ref.** |
| --- | --- | --- | --- | --- |
| Cyclophosphamide | L-TACB Lymphoma; euthymic vs athymic nude rats | Antimetastatic efficacy was reduced in athymic nude rats. | Treg-targeting or immune modulation is insufficient without cytotoxic T-cell participation. | ^11^ |
| Cyclophosphamide | EL4 Lymphoma; euthymic vs immunosuppressed rats | Durable control and resistance to subsequent tumor challenge were reduced in immunosuppressed rats. | Immune surveillance and memory contribute substantially to cyclophosphamide efficacy. | ^12^ |
| Cyclophosphamide + Gemcitabine | CT26 Colorectal Carcinoma; immune-replete vs athymic nude and/or T-cell-depleted mice | Combination efficacy was diminished in immunodeficient comparators. | Concurrent Treg/MDSC depletion requires adaptive effector function for full therapeutic benefit. | ^13^ |
| Gemcitabine | 4T1 Mammary Carcinoma; immune-replete vs athymic nude and/or T-cell-depleted mice | Suppressive-myeloid reduction did not translate into full antitumor benefit in immunodeficient comparators. | Myeloid depletion alone does not substitute for T-cell-mediated tumor killing. | ^14^ |
| 5-fluorouracil | CT26 Colorectal Carcinoma; immune-replete vs athymic nude (nu/nu) mice and/or T-cell-depleted mice | MDSC depletion enhanced antitumor immunity in immune-replete hosts, but downstream immune benefit was not reproduced in T-cell-deficient comparators. | Relief of myeloid suppression requires a downstream T-cell compartment to generate durable efficacy. | ^8^ |
| Mitoxantrone | CT26 Colorectal Carcinoma and MCA205 Fibrosarcoma; immune-replete vs athymic nude and/or T-cell-depleted mice | Immunogenic efficacy was lost or markedly reduced in T-cell-deficient comparators despite preserved cancer-cell death. | Calreticulin-linked ICD requires CD8⁺ T-cell engagement for full therapeutic expression. | ^15^ |
| Gemcitabine | TC-1 Lung Carcinoma, AB12 Malignant Mesothelioma, Lewis Lung Carcinoma (LLC), and B16-F10 Melanoma; immune-replete vs athymic nude and SCID mice | Immunomodulatory effects were observed, but full adaptive antitumor clearance was limited in severely immunodeficient hosts. | Reduction of Tregs/MDSCs is not sufficient when T-cell-mediated tumor elimination is absent. | ^16^ |

**References**

1	Liu, P. *et al.* Crizotinib-induced immunogenic cell death in non-small cell lung cancer. *Nature communications* 10, 1486 (2019).

2	Naito, T. *et al.* High-dose cyclophosphamide induces specific tumor immunity with concomitant recruitment of LAMP1/CD107a-expressing CD4-positive T cells into tumor sites. *Cancer letters* 366, 93–99 (2015).

3	Čulo, F., Klapan, I. & Kolak, T. The influence of cyclophosphamide on antitumor immunity in mice bearing late-stage tumors. *Cancer Immunology, Immunotherapy* 36, 115–122 (1993).

4	Moore, M. & Williams, D. E. Contribution of host immunity to cyclophosphamide therapy of a chemically‐induced murine sarcoma. *International journal of cancer* 11, 358–368 (1973).

5	Evans, R. Further observations on the effect of cyclophosphamide on intratumor and peripheral leukocyte levels. *The American Journal of Pathology* 99, 667 (1980).

6	Palomares, T., Bilbao, P., Alonso-Varona, A. & Barberá-Guillem, E. The addition of interleukin-2 to cyclophosphamide therapy can facilitate tumor growth of B16 melanoma. *Cancer Immunology, Immunotherapy* 40, 292–298 (1995).

7	Reissmann, T., Voegeli, R., Pohl, J. & Hilgard, P. Augmentation of host antitumor immunity by low doses of cyclophosphamide and mafosfamide in two animal tumor models. *Cancer Immunology, Immunotherapy* 28, 179–184 (1989).

8	Taniura, T. *et al.* Immunogenic chemotherapy in two mouse colon cancer models. *Cancer science* 111, 3527–3539 (2020).

9	Guan, Y. *et al.* FOLFOX chemotherapy ameliorates CD8 T lymphocyte exhaustion and enhances checkpoint blockade efficacy in colorectal cancer. *Frontiers in oncology* 10, 586 (2020).

10	Limagne, E. *et al.* Trifluridine/tipiracil plus oxaliplatin improves PD-1 blockade in colorectal cancer by inducing immunogenic cell death and depleting macrophages. *Cancer immunology research* 7, 1958–1969 (2019).

11	Matar, P., Rozados, V. R., Roggero, E. A., Bonfil, R. D. & Scharovsky, O. G. Modulation of the antimetastatic effect of a single low dose of cyclophosphamide on rat lymphoma. *Tumor biology* 19, 69–76 (1998).

12	Vidović, D., Marušić, M. & Čulo, F. Interference of anti-tumor and immunosuppressive effects of cyclophosphamide in tumor-bearing rats: Analysis of factors determining resistance or susceptibility to a subsequent tumor challenge. *Cancer Immunology, Immunotherapy* 14, 36–40 (1982).

13	Tongu, M. *et al.* Metronomic chemotherapy with low-dose cyclophosphamide plus gemcitabine can induce anti-tumor T cell immunity in vivo. *Cancer immunology, immunotherapy* 62, 383–391 (2013).

14	Le, H. K. *et al.* Gemcitabine directly inhibits myeloid derived suppressor cells in BALB/c mice bearing 4T1 mammary carcinoma and augments expansion of T cells from tumor-bearing mice. *International immunopharmacology* 9, 900–909 (2009).

15	Obeid, M. *et al.* Calreticulin exposure dictates the immunogenicity of cancer cell death. *Nature medicine* 13, 54–61 (2007).

16	Suzuki, E., Sun, J., Kapoor, V., Jassar, A. S. & Albelda, S. M. Gemcitabine has significant immunomodulatory activity in murine tumor models independent of its cytotoxic effects. *Cancer biology & therapy* 6, 880–885 (2007).
